# Design and Analysis of a Quasi-Yagi Antenna for an Indoor Location Tracking System

**DOI:** 10.3390/s18124246

**Published:** 2018-12-03

**Authors:** Sun-Woong Kim, Sun-Kuk Noh, Ho-Gyun Yu, Dong-You Choi

**Affiliations:** 1 IT Research Institute, Chosun University, Gwangju 61452, Korea; woongskim1@naver.com; 2 SW Convergence Education Institute, Chosun University, Gwangju 61452, Korea; nsk7078@chosun.ac.kr; 3 Department of Information and Communication Engineering, Graduate School, Chosun University, Gwangju 61452, Korea; 1030ghrbs@naver.com

**Keywords:** quasi-yagi antenna, indoor location tracking system, IR-UWB (Impulse-Radio Ultra Wideband) radar, parasitic director

## Abstract

In this paper, a quasi-Yagi antenna for an indoor location tracking system is proposed. The performance of the proposed antenna was verified by testing it using an indoor location tracking system. To improve the bandwidth and gain, two parasitic directors were added near the dipole. The performance verification of the proposed antenna is explained, along with a performance comparison of the VSWR (voltage standing wave ratio) radiation pattern and the realized gain. The proposed antenna was connected to an NVA-R661 module of Xethru Inc. for indoor location tracking. The proposed antenna exhibited a wide bandwidth of 4.36 GHz by satisfying a VSWR ≤ 2 from 5.03 to 9.39 GHz, the maximum gain was 6.46 dBi in the 8 GHz band. The radiation pattern exhibited a good directivity characteristic within the proposed band. The location tracking result of a moving target clearly describes the route of the target along a moving line.

## 1. Introduction

While GPS (global positioning system), the main outdoor location tracking system, has shown high performance and precision, various methods for an indoor location tracking system have been studied due to the absence of a clear solution [1,2]. 

Conventional indoor location tracking solutions and security solutions have various problems. Good performance can be difficult to achieve in image-based systems if sight is covered by obstacles such as fog and smoke. This is also true in the case of the infrared sensors because they are highly sensitive to temperature and humidity [3].

In this paper, an antenna for an indoor location tracking system is proposed. The performance of this antenna was verified by connecting it to the impulse radar-based technology of an NVA-R661 module of Xethru Inc. (Oslo, Norway). The NVA-R661 module, which is an IR-UWB radar system, has a wide bandwidth of 6.0 to 10.2 GHz [4]. 

Typically, the IR-UWB radar system has high spatial resolution because it uses a short pulse signal with a bandwidth above 500 MHz or a fractional bandwidth greater than 0.2 [5]. Therefore, it has higher penetrability than other indoor security solutions, as well as precise location tracking. 

Indoor location tracking systems based on IR-UWB radars have been implemented using various antennas, such as monopole antennas [6,7], patch antennas [8], Yagi-type antennas [9,10,11,12,13,14], and tapered-slot antennas [15].

The proposed antenna was fabricated as a quasi-Yagi type and, to improve the bandwidth and gain, two parasitic directors were added near the dipole. The fabricated quasi-Yagi antenna was connected to an NVA-R661, thus verifying the validity of the indoor location tracking systems. 

This paper is organized as follows. In Section 2, the design and fabrication of the proposed quasi-Yagi antenna is described. In Section 3, the validity of the indoor location tracking system is verified by connecting the antenna to the NVR-R661 module. In Section 4, the conclusion is provided.

## 2. Fabrication and Analysis of the Antenna

### 2.1. Design and Simulation of the Antenna 

**Figure 1:**
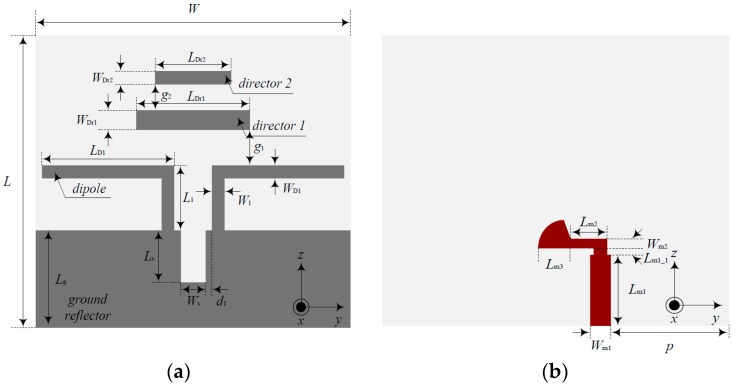
Structure of the quasi-Yagi antenna: (**a**) top layer, and (**b**) bottom layer.

The complete structure of the proposed quasi-Yagi antenna is shown in Figure 1. It was printed on a TRF-45 substrate, which had a dielectric constant of 4.5, a loss tangent of 0.0035, and a thickness of 0.61 mm. 

A dipole (*L*_D1_, *W*_D1_) for the radiation of the antenna was placed on the top layer and two parasitic strip directors (*L*_Dr1_, *L*_Dr2_, *W*_Dr1_, *W*_Dr2_) were added to increase the bandwidth and the gain. A 50 Ω feed line for the impedance matching of the antenna was placed on the bottom layer according to parameters *L*_m1_, *L*_m2_, *W*_m1_, and *W*_m2_. The ground reflector was connected to the dipole to improve the directivity of the antenna according to parameter *L*_S_. Then, the design parameters of the proposed quasi-Yagi antenna were as follows: *L* = 32.7 mm, *W* = 33 mm, *L*_1_ = 6.4 mm, *W*_1_ = 1.6 mm, *L*_D1_ = 14 mm, *W*_D1_ = 1.4 mm, *L*_Dr1_ = 10 mm, *W*_Dr1_ = 2 mm, *L*_Dr2_ = 7.5 mm, *W*_Dr2_ = 1.4 mm, *L*_S_ = 5.7 mm, *W*_S_ = 2.6 mm, *L*_m1_ = 7.5 mm, *L*_m1_1_ = 0.5 mm, *W*_m1_ = 2 mm, *L*_m2_ = 3.2 mm, *W*_m2_ = 1 mm, *L*_m3_ = 3.2 mm, *L*_g_ = 10.7 mm, *d*_1_ = 0.7 mm, *g*_1_ = 3.8 mm, *g*_2_ = 3 mm, and *p* = 11.3 mm. 

**Figure 2:**
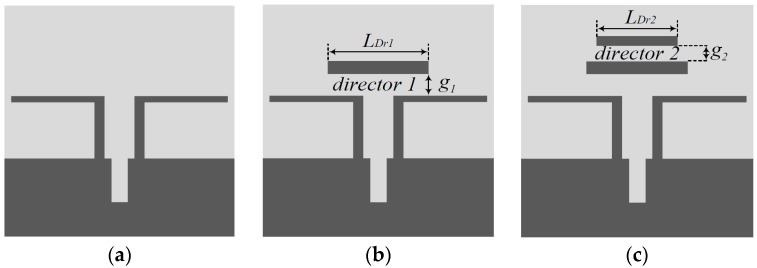
Three steps of the proposed antenna design process: (**a**) Antenna-1, (**b**) Antenna-2, and (**c**) Antenna-3.

The proposed antenna was analyzed in three steps as shown in Figure 2. 

As shown in Figure 2, Antenna-1 is a basic quasi-Yagi antenna without the parasitic directors. In Antenna-2 and Antenna-3, parasitic strip directors were added to achieve a wide bandwidth impedance matching and high gain near the dipole.

**Figure 3:**
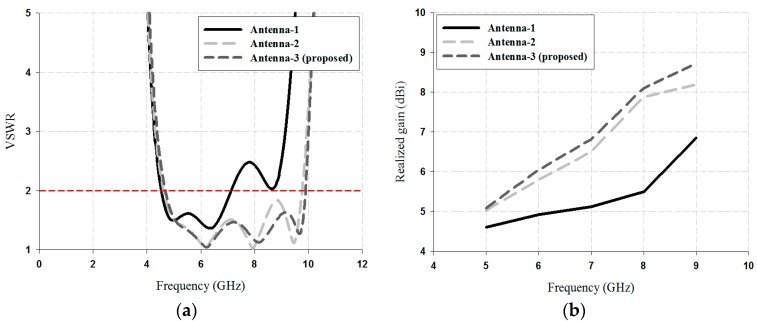
Performance analysis of the three steps: (**a**) impedance bandwidth of VSWR, and (**b**) antenna gain.

The VSWR ≤ 2 impedance bandwidth and the realized gain simulation results of the three steps, were analyzed using HFSS (High Frequency Simulation Software) Version 12, and are shown in Figure 3. 

As shown in Figure 3, Antenna-1 exhibited good impedance matching by satisfying a VSWR ≤ 2 from 4.54 to 7.15 GHz. The realized gain results of Antenna-1 were 4.61 dBi at 5 GHz, 4.92 dBi at 6 GHz, 5.12 dBi at 7 GHz, 5.50 dBi at 8 GHz, and 6.85 dBi. In Antenna-2, a parasitic director was added to improve the bandwidth and the high gain, and the impedance bandwidth converged with a VSWR ≤ 2 from 4.60 to 9.77 GHz. The realized gain results of Antenna-2 were 5.03 dBi at 5 GHz, 5.80 dBi at 6 GHz, 6.50 dBi at 7 GHz, 7.88 dBi at 8 GHz, and 8.19 dBi. Finally, Antenna-3 exhibited a wider bandwidth result and a higher gain than Antenna-2 and Antenna-1. The impedance bandwidth converged with a VSWR ≤ 2 from 4.67 to 9.89 GHz and the realized gain results were 5.09 dBi at 5 GHz, 6.05 dBi at 6 GHz, 6.82 dBi at 7 GHz, 8.10 dBi at 8 GHz, and 8.72 dBi. 

**Figure 4:**
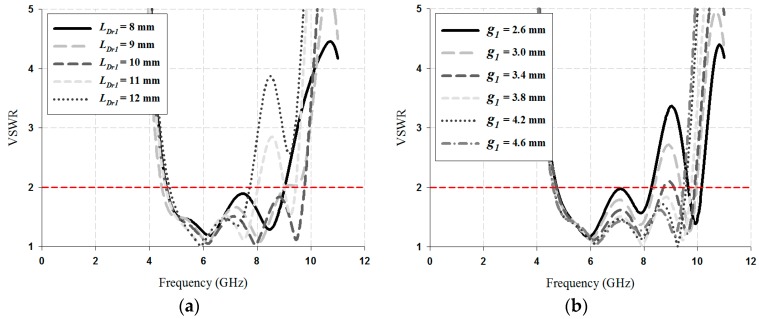
VSWR results simulated for director-1: (**a**) *L_Dr_*_1_, and (**b**) *g*1.

**Figure 5:**
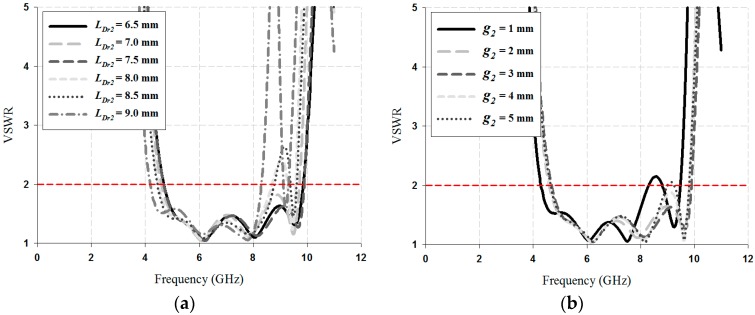
VSWR results simulated for director-2 (**a**) *L_Dr2_*, (**b**) *g*2.

The VSWR ≤ 2 impedance bandwidth results simulated for the values of *L_Dr_*_1_, *L_Dr_*_2_, *g*_1_, and *g*_2_ of the parasitic directors are shown in Figure 4 and Figure 5. 

As shown in Figure 4, as a result of varying the director-1, the variation of the impedance bandwidth is obvious. The bandwidth results of the value *L_Dr_*1 for the director-1 were 4.42 GHz for 8 mm, 4.45 GHz for 9 mm, 5.15 GHz for 10 mm, 3.45 GHz for 11 mm, and 3.01 GHz for 12 mm. For the case of the value *g*_1_, the bandwidth results were 3.55 GHZ for 2.6 mm, 3.69 GHz for 3 mm, 4.04 GHz for 3.4 mm, 5.15 GHz for 3.8 mm, 4.97 GHz for 4.2 mm, and 4.89 GHz for 4.6 mm. Therefore, the values of *L_Dr_*1 and *g*1 for the director-1 were chosen as 10 mm and 3.8 mm, respectively.

As shown in Figure 5, as a result of varying the director-2, the VSWR was higher in the 8 to 9 GHz band. VSWR was closer to 1 when the *L_Dr_*_2_ and *g*_2_ for director-2 were 7.5 mm and 3 mm, respectively; in addition, the bandwidth was 5.22 GHz.

**Figure 6:**
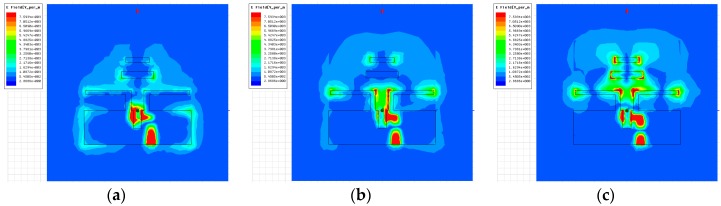
Current distribution results of the proposed antenna: (**a**) 5 GHz, (**b**) 7 GHz, and (**c**) 9 GHz.

The current distribution simulation results of the proposed antenna are shown in Figure 6.

As shown in Figure 6, due to the ground reflector connected to the dipole, the current was suppressed in the –*z*-axis, and concentrated in the +*z*-axis. Therefore, director-1 and director-2 contributed to the radiation at the high bandwidth (8–9 GHz band) by distributing the current. Furthermore, the current distribution in Figure 6c was concentrated in director-1 and director-2, and contributed to the radiation in the high band. 

**Figure 7:**
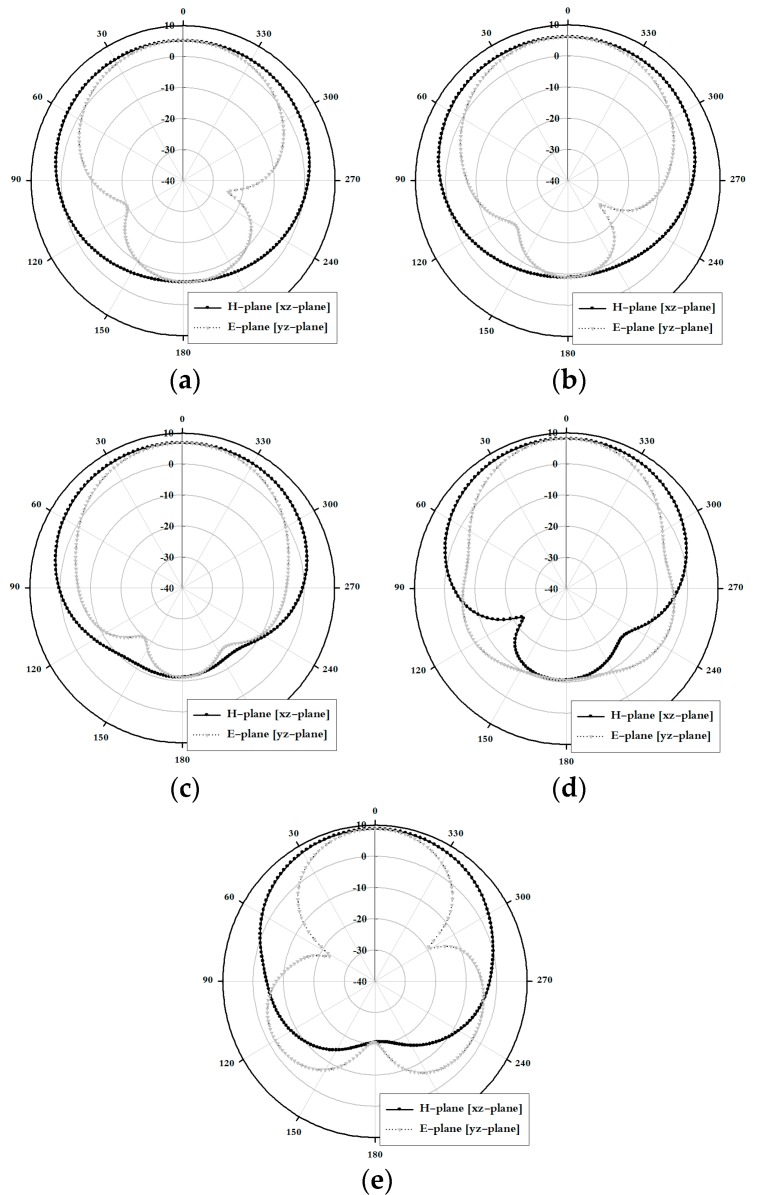
Simulated radiation pattern results of the proposed antenna: (**a**) 5 GHz, (**b**) 6 GHz, (**c**) 7 GHz, (**d**) 8 GHz, and (**e**) 9 GHz.

The simulated radiation pattern results along the xz-plane (H-plane) and the yz-plane (E-plane) of the proposed antenna are shown in Figure 7.

As shown in Figure 7, the maximum gain was concentrated along the +*z*-axis within the proposed band and exhibited good directional characteristics. 

### 2.2. Measurement Analysis of the Fabricated Antenna

**Figure 8:**
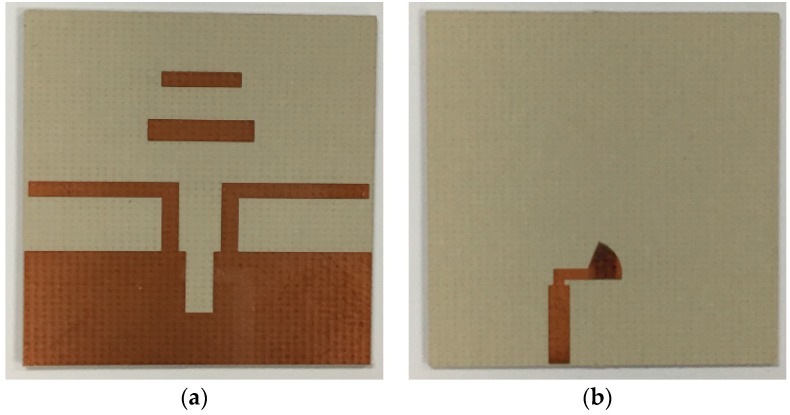
Photograph of the fabricated antenna: (**a**) top layer, and (**b**) bottom layer.

The design of the fabricated antenna was based on the simulation, as shown in the photographs of Figure 8.

**Figure 9:**
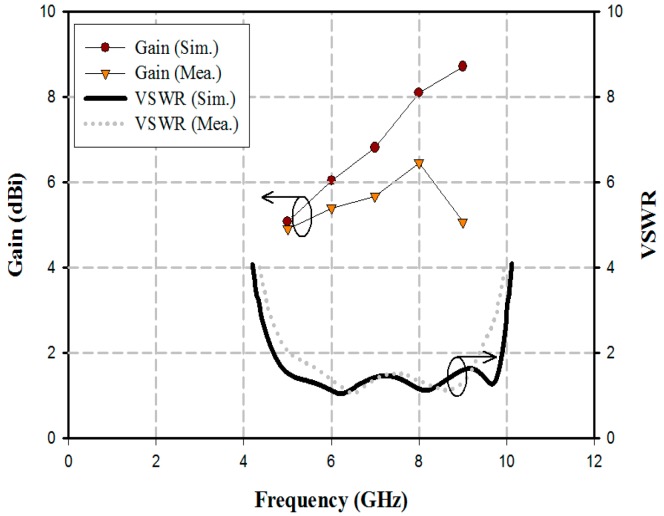
VSWR and realized gain of the fabricated antenna.

The VSWR ≤ 2 impedance bandwidth and the realized gain measured results of the fabricated antenna are shown in Figure 9. 

As shown in Figure 9, the fabricated antenna exhibited a wide bandwidth of 4.36 GHz by satisfying a VSWR ≤ 2 from 5.03 to 9.39 GHz. Furthermore, the measured realized gain results were 4.91 dBi at 5 GHz, 5.41 dBi at 6 GHz, 5.68 dBi at 7 GHz, 6.46 dBi at 8 GHz, and 5.08 dBi at 9 GHz. Simulated and measured results of the realized gain agree well for 5 to 6 GHz, but not for differences at higher frequencies above 6 GHz that may come from the fabrication error, etching process, the influence of the SMA connector, coaxial cables on the radiation characteristics, and the measured conditions and different environmental factors. However, the gain of the antenna was not hampering the detection of the position of moving object within a limit range of 1 to 4 m.

**Figure 10:**
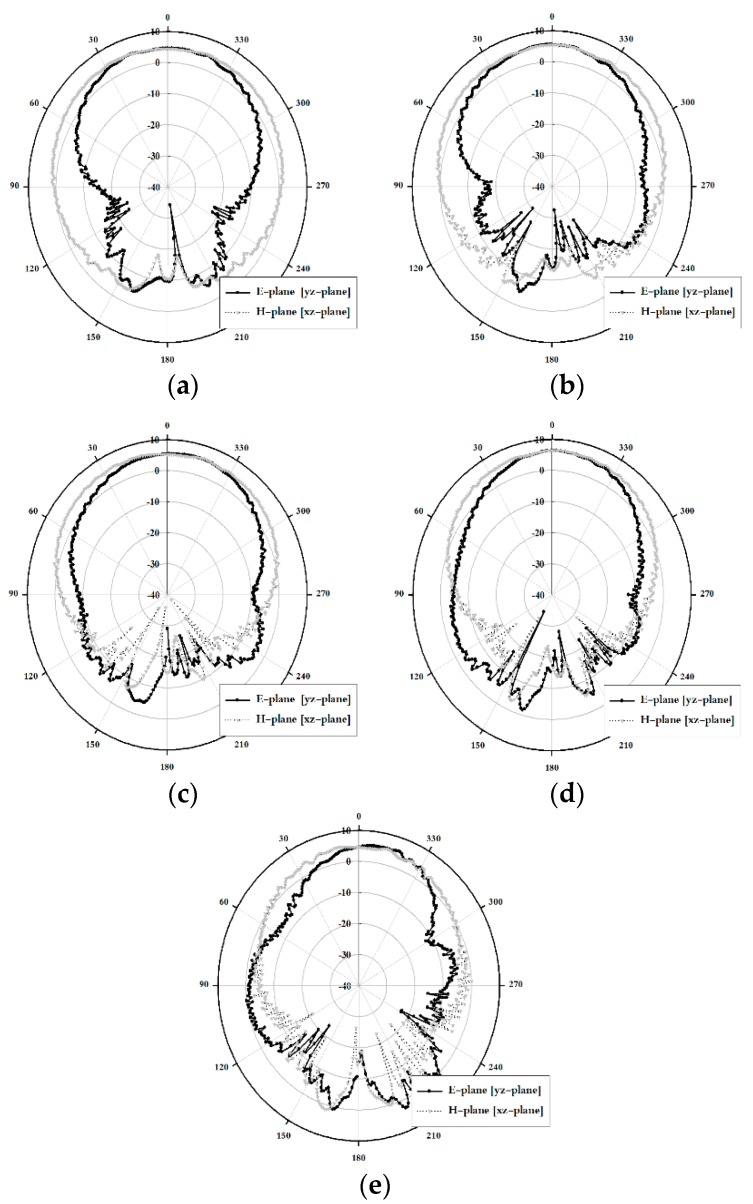
Measured radiation pattern results of the proposed antenna: (**a**) 5 GHz, (**b**) 6 GHz, (**c**) 7 GHz, (**d**) 8 GHz, and (**e**) 9 GHz.

The measurement radiation pattern results along the xz-plane (H-plane) and the yz-plane (E-plane) of the proposed antenna are shown in Figure 10.

As shown in Figure 10, the maximum gain was concentrated along the +*z*-axis within the proposed band and exhibited good directional characteristics. 

## 3. Measurement Platform for Indoor Location Tracking System

### 3.1. Radar Set-Up 

**Figure 11:**
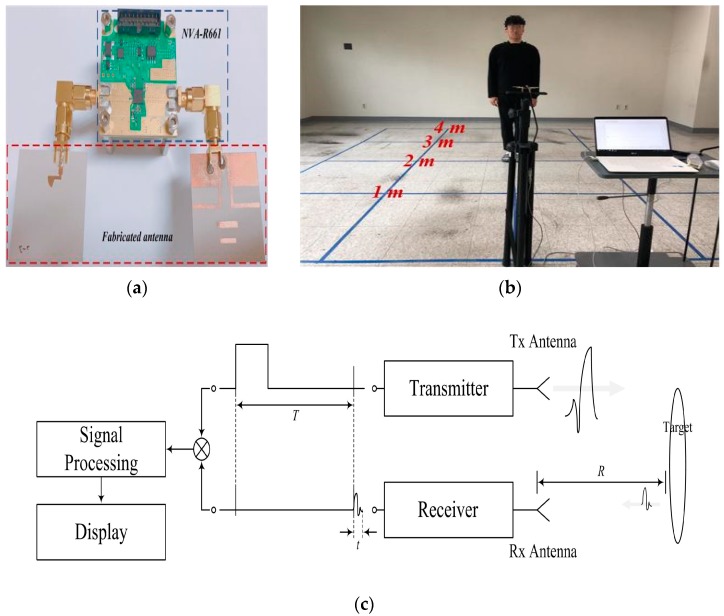
Measurement platform for indoor location tracking system: (**a**) radar set-up, (**b**) experimental set-up, and (**c**) block diagram of the measurement platform.

As shown in Figure 11, an indoor location tracking system was set up for verifying the practicality of the fabricated antenna. The radar module for the indoor location tracking system was the NVA-R661 of Xethru Inc.

As shown in Figure 11, the basic radar configuration consisted of a transmitter and a receiver. The measured distance *R* of the target is given by:

(1) $$R=\frac{ct}{2}$$

where *R* is the distance of the target, *t* is the time difference between the transmitted signal and the received signal, and *c* is the velocity of light [16]. The measured data is analyzed through the signal processing and the distance *R* is displayed. 

**Figure 12:**
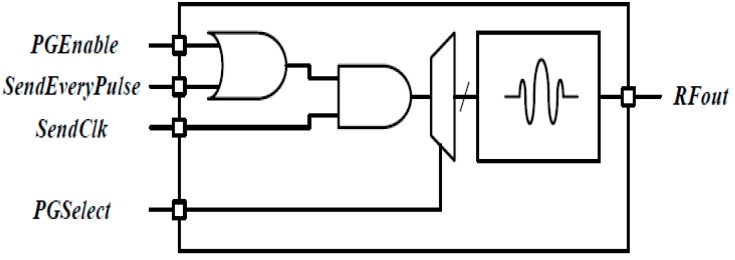
Block diagram of the transmitter.

The X2 chip of NVA-R661 module generates a high-order Gaussian impulse signal, and it transmits the impulse signal that has several GHz bandwidth in order of nanoseconds. Figure 12 shows the transmitter block diagram of NVA-R661 provide by Xethru Inc. 

**Figure 13:**
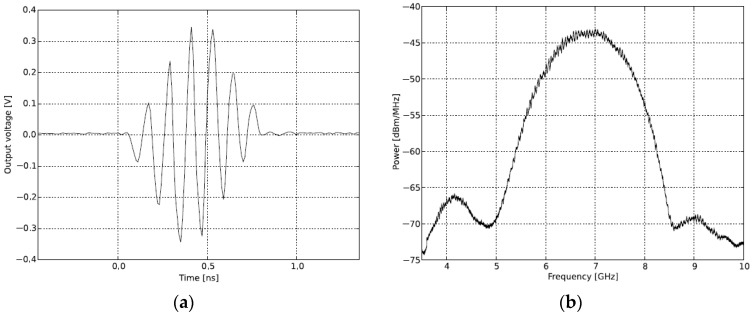
Frequency band and transmitting signal of IR-UWB radar: (**a**) transmitting signal in the time domain, and (**b**) transmitting signal in the frequency domain.

As shown in Figure 12, *PGSelect* and *SendEveryPulse* are user configurable functions and the center frequency of the impulse signal is set-up through the *PGSelect* segment. The *PGSelect* segment has setting numbers from 0 to 11, where a higher number of *PGSelect* corresponds the higher center frequency. In our experiment, setting number 5 of a *PGSelect* segment was selected, and the transmitting signal is shown in Figure 13.

As shown in Figure 13, the transmitting signal was observed to be a short pulse in order of nanoseconds, having a center frequency in the 6.8 GHz band. The detailed description of each function for block diagram is provided in the technical document of Xethru Inc., and this paper skips them. The impulse signal of the proposed radar system was chosen with setting number 5 of *PGSelect* that has a center frequency at 6.8 GHz, and it is close to the center frequency of the proposed antenna [17].

### 3.2. Signal Processing Set-Up 

**Figure 14:**
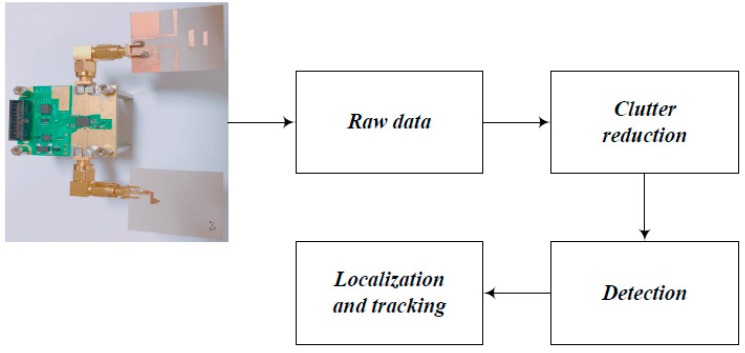
Signal processing configuration for the indoor location tracking system.

The signal processing configuration for the indoor location tracking system is shown in Figure 14.

As shown in Figure 14, the signal processing configuration consisted of clutter reduction, detection, localization, and tracking. The signal reflected from the target is given by:

(2) $$r_{i}=r_{c,i}+r_{t,i}+r_{n}$$

where *r_i_* is the *i*-th received signal, which consists of the clutter signal *r_c_*_,*i*_, the target signal *r_t_*_,*i*_, and noise *r_n_*. 

In the clutter reduction step, the primary goal is to remove the clutter signal from the raw data. This signal can be found using SVD (singular value decomposition) to the target signal *r_t_*_,*i*_. The *n* signal received can form a matrix *R* with *R* = [*R*_1_, *R*_2_, ..., *R*_n_]^t^. The matrix *R* can be separated from the clutter signal *R_C_*, target signal *R_T_*, and noise *N*. The matrix *R* is given by:

(3) $$R=R_{C}+R_{T}+N\;R=\left[\begin{array}{c} R_{1}\\ R_{2}\\ \vdots \\ R_{n} \end{array}\right],\;R_{C}=\left[\begin{array}{c} R_{c,\;1}\\ R_{c,\;2}\\ \vdots \\ R_{c,\;n} \end{array}\right],\;R_{T}=\left[\begin{array}{c} R_{T,\;1}\\ R_{T,\;2}\\ \vdots \\ R_{T,\;n} \end{array}\right],\;N=\left[\begin{array}{c} N_{1}\\ N_{2}\\ \vdots \\ N_{n} \end{array}\right]$$

The matrix *R* received through SVD is separated by the signals that has the eigenvalue and the eigenvector. Where, the eigenvalues are the weights of the linearly independent signals, and the eigenvectors are the temporal and spatial variation values. To remove the clutter signal, *R* is decomposed using *R* = *USV^T^* through SVD:

(4) [U,S,V]=SVD(R)

where *R* is an *m × n* matrix of the scanning data; here, *m* represents the time frame and n represents the target position. *U* is the orthogonal matrix of *m* × *m*, *V* is the orthogonal matrix of *n* × *n*, and *S* is the diagonal matrix of *m* × *n*. The columns of matrix *U* is the left singular vector, and the columns of matrix *V* is the right singular vector. When the rank r of *R* satisfies r = *n* < *m*, meaning that within the observable time frame, the target positions are always within the frame boundary. SVD of *R* is obtained using:

(5) $$R=USV^{T}=\left[\delta_{1}\delta_{2}\delta_{3}\cdots \delta_{n}\right]\left[\begin{array}{ccccccc} v_{1} & 0 & \cdots & 0 & 0 & \cdots & 0\\ 0 & v_{2} & & \vdots & \vdots & & \vdots \\ \vdots & & \ddots & 0 & & & \\ 0 & \cdots & 0 & v_{n} & 0 & \cdots & 0 \end{array}\right]\left[\begin{array}{c} \alpha_{1}^{T}\\ \alpha_{2}^{T}\\ \alpha_{m}^{T} \end{array}\right]$$

The coefficient of *R* is r, which can be expressed using a group of sampling frames given by Equations (6) and (7) using the above singular value decomposition:

(6) $$R=v_{1}\left(\begin{array}{c} \vdots \\ \delta_{1}\\ \vdots \end{array}\right)\left(\cdots \;\alpha_{1}^{T}\cdots \right)+v_{2}\left(\begin{array}{c} \vdots \\ \delta_{2}\\ \vdots \end{array}\right)\left(\cdots \;\alpha_{2}^{T}\cdots \right)+\cdots +v_{r}\left(\begin{array}{c} \vdots \\ \delta_{r}\\ \vdots \end{array}\right)\left(\cdots \;\alpha_{r}^{T}\cdots \right)$$

(7) $$R=\sum_{i=1}^{r}v_{i}\delta_{i}\alpha_{i}{}^{T}$$

where, δ*_i_* and α*_i_* are the *i-*th eigenvectors to the left and right of *R*, and *v_i_* represents the *i*-th singular value of *R*. The matrix can be reconstructed using the eigenvalues that are relatively dominant among the eigenvalues obtained using SVD. The reconstructed matrix is approximated, and this approximation is called the low rank approximation. Using the low rank approximation, *R* is approximated to matrix *R*_j_ with rank *j*:

(8) $$R_{j}=\sum_{i=1}^{j}v_{i}\delta_{i}\alpha_{i}{}^{T}$$

The approximated *R*_j_ is the matrix reconstructed as a temporal principal component signal through the priority of the eigenvalue. Therefore, the approximated *R*_j_ can be seen as the clutter signal matrix *R*_c_. In the above Equation (3), we can obtain the signal matrix *R*_T_ of the target when the clutter signal of matrix *R*_c_ are subtracted from a received signal of matrix *R*. The target signal matrix *R_T_* is obtained as follows from Reference [18]:

(9) $$R_{T}+N=R-R_{C}=R-R_{j}.$$

**Figure 15:**
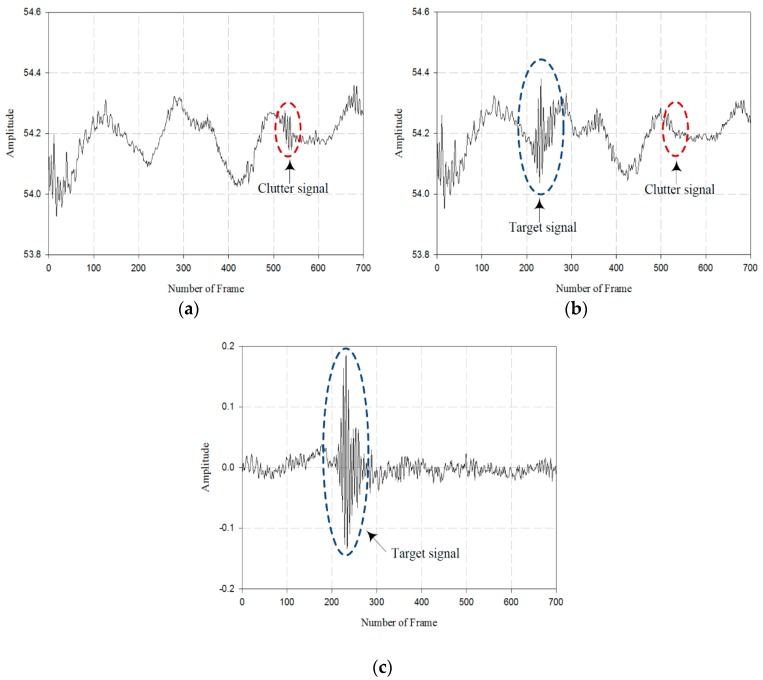
Target signal detection process through SVD: (**a**) signal received without target, (**b**) signal received with target, and (**c**) target signal that removed clutter.

The target signal detection process through SVD is shown in Figure 15. 

As shown in Figure 15, the clutter signals detected in the indoor experiment setup include the walls, pillars, and the other obstacles. The wall was located behind the target, and the clutter signals reflected by the wall are little and there was no change of signal in the preceding time frame. However, the target signal movement was large and is changing over in time. By reconstructing the received signal matrix, the clutter signals with no change over the time were considered to be the clutter signals. The target signal obtained through SVD is shown in Figure 15c. 

After the clutter reduction algorithm was applied through the detection process, the location information of the target was obtained. In the detection process, the signal of the target of the maximum values is detected through cross-correlation in between the received signal and the transmitted signal. 

**Figure 16:**
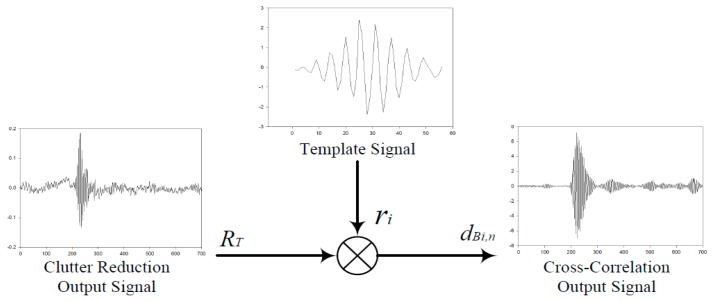
Target signal detection process through cross-correlation.

In order to detect the signal through cross-correlation, the following equation is used:

(10) $$d_{Bi,n}\left(t\right)=R_{T}\left(t\right)\times r_{i}\left(t\right)=\int R\left(\tau -t\right)s\left(\tau \right)d\tau$$

where *d_Bi,n_* indicates the results of the cross-correlation between the output signal after the clutter reductions and the template signal *r_i_*. The template signal is the transmitted signal generated in the X2 chipset. The target signal detection process through cross-correlation is shown in Figure 16. 

As shown in Figure 16, a signal *d_Bi,n_* was the cross-correlation output signals value that is found using the correlation analysis in between the clutter reduction output signal *R_T_* and the template signal *r_i_*. Therefore, through the maximum values of cross-correlation results, the location of the target was estimated. In the localization and tracking step, the distance of the target was determined by using the ToA (time-of-arrival) of the measured target signal [19,20,21,22].

### 3.3. Target Tracking Measurement and Antenna Verification

**Figure 17:**
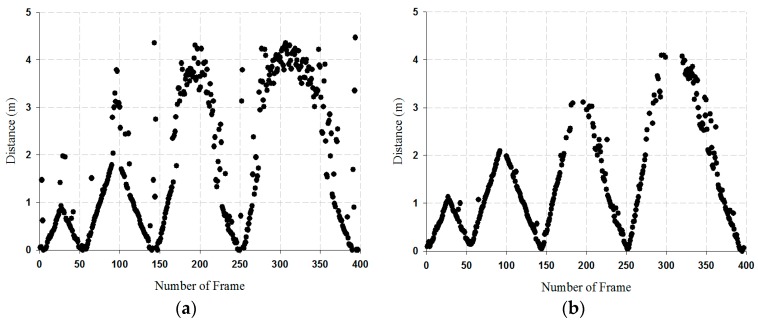
Location tracking result of the moving target: (**a**) AVG results, and (**b**) SVD results.

To verify the performance of the clutter reduction algorithm, it is compared with SVD and AVG (average filter). In order to track the location of the single target, the distance was measured based on 400 data points moving from 1 to 4 m within the radar range. The location tracking result of the moving target for SVD and AVG algorithm is shown in Figure 17.

As shown in Figure 17, a single target walked back and forth from 1 to 4 m in units of 1 m. The results of the AVG filter were observed with very low resolution. In the case of SVD, it was observed to have a good resolution for 0 to 2 m, but some error existed while analyzing in the distance from 3 to 4 m. The reason is that the received signal became weaker as the distance increased. The RMSE (root mean square error) that resulted from the algorithm of the SVD and AVG filter was 0.2405 and 0.7820, respectively. From the experimental results, we see that the RMSE result of the SVD was lower than the AVG filter with the conclusion that SVD observed the better resolution. 

**Figure 18:**
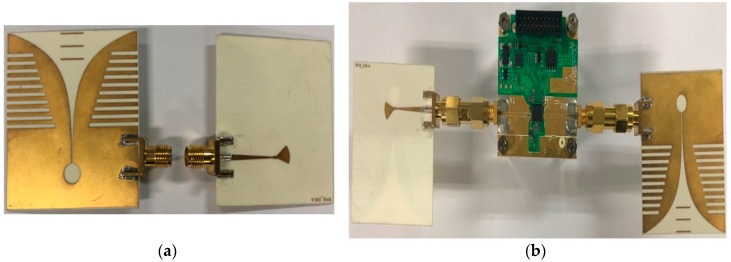
Measurement platform of the commercial antenna: (**a**) structure, and (**b**) radar set-up.

To verify the validity performance of the proposed antenna, comparison with the proposed antenna and the commercial antenna was made. The structure and radar set-up of the commercial antenna is shown in Figure 18 [23].

As shown in Figure 18, the type of the commercial antenna is a Vivaldi-LA antenna, and the overall size was about 50 × 50 × 2 mm^3^. The specification of the commercial antenna is listed in the Table 1. 

**Figure 19:**
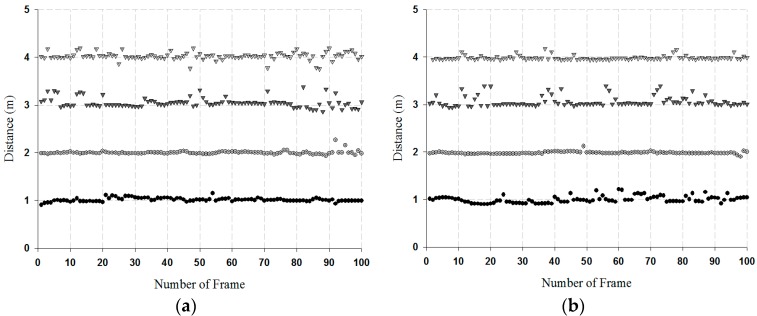
Measurement results by distance for a fixed single target: (**a**) results of proposed antenna, and (**b**) results of the commercial antenna.

The proposed antenna and the commercial antenna detected a single target in the indoor position environment. A single target was measured from the distance of 1 to 4 m that was fixed in units of 1 m, and the measurement results are shown in Figure 19. 

As shown in Figure 19, the measurement results of the proposed antenna and the commercial antenna were observed with some errors in the distance of 3 to 4 m. This was because the received signal became weaker as the distance increased. The proposed antenna and the commercial antenna was compared through the RMSE, and its values are listed in Table 2. 

As shown in Table 2, the proposed antenna and the commercial antenna had similar performances, but the proposed antenna had the advantage of being compact, less weight, and portable compared with the other commercial antenna.

The proposed antenna was compared with antennas applied in similar systems, as described in Table 3. 

As shown in Table 3, the proposed antenna, while exhibiting similar results in bandwidth and gain, had the advantage of being smaller than the other antennas. Furthermore, the performance of the proposed antenna was verified by connecting it to the indoor location tracking system. 

## 4. Conclusions

The fabricated quasi-Yagi antenna was proposed for an indoor location tracking system. By adding two parasitic directors near the dipole, the fabricated antenna improved its impedance bandwidth and its realized gain. Furthermore, the performance of the proposed antenna was verified by connecting it to the NVA-R661 for an indoor location tracking system. 

The fabricated antenna showed a bandwidth of 4.36 GHz by satisfying a VSWR ≤ 2 from 5.03 to 9.39 GHz and the measured realized gain was 4.91 dBi at 5 GHz, 5.41 dBi at 6 GHz, 5.68 dBi at 7 GHz, 6.46 dBi at 8 GHz, and 5.08 dBi at 9 GHz. The measured radiation pattern result exhibited good directional characteristics. Finally, the fabricated antenna could clearly describe the movement of a target that moved forward and backward four times inside the imaging area along the moving line in the range from 1 to 4 m. 

## Figures and Tables

**Table 1:** Comparisons of the proposed antenna and reference antennas.

Type	Vivaldi-LA
Gain	8 dBi
Polarization	Linear
Impedance	50 Ω
Size	50 × 50 × 2 mm^3^
Connector	SMA

**Table 2:** RMSE results of the proposed antenna and the commercial antenna.

Distance (m)	RMSE	
	Proposed Antenna	Commercial Antenna
1	0.0406	0.0716
2	0.0367	0.0295
3	0.1157	0.1287
4	0.0846	0.0469

**Table 3:** Comparisons of the proposed antenna and reference antennas.

Antenna	Ref. [15]	Ref. [24]	Ref. [25]	This Work
Antenna type	Tapered slot	Quasi-Yagi	Quasi-Yagi	Quasi-Yagi
Size (mm)	130 × 70	36 × 35	90 × 140	32.7 × 33
Bandwidth (GHz)	0.64–6	3.8–10.3	1.59–3.64	5.03–9.39
Gain (dBi)	-	7 dBi	7.4 dBi	6.46 dBi
Location tracking (m)	1–9	-	-	1–4

## References

[1] Yoon D.Y. (2012). A Design of the Impulse Radar Digital Signal Processing for the Precise Location Positioning in IR-UWB. Master’s Thesis.

[2] Immoreev I.J. (2004). Ultra-wideband Systems. Features and Ways of Development.

[3] Kim J.H. (2013). A Design of the Ranging Algorithm for Multi-Targets Using IR-UWB. Master’s Thesis.

[4] XETHRU UWB Module. https://www.xethru.com/.

[5] Kim K.Y., Kang E.K., Kim J.W., Ra K.W. (2014). A Study and Design of Beam Scanning Array Antenna using IR-UWB. J. Inst. Electron. Inf. Eng..

[6] Zarrabi F.B., Mansouri Z., Gandji N.P., Kuhestani H. (2016). Triple-Notch UWB Monopole Antenna with Fractal Koch and T-Shaped Stub. Int. J. Electron. Commun..

[7] Bakariya P.S., Dwari S., Sarkar M. (2015). Triple Band Botch UWB Printed Monopole Antenna with Enhanced Bandwidth. Int. J. Electron. Commun..

[8] Rabbani M.S., Ghafouri-Shiraz H. (2017). Accurate Remote Vital Sign Monitoring with 10 GHz Ultra-Wide Patch Antenna Array. Int. J. Electron. Commun..

[9] Wu J., Zhao Z., Nie Z., Liu Q.H. (2014). Bandwidth Enhancement of a Planar Printed Quasi-Yagi Antenna with Size Reduction. IEEE Trans. Antennas Propag..

[10] Yang D., Qu J., Zhao Z., Liu S., Nie Z. (2016). Planar Quasi-Yagi Antenna with Band Rejection Based on Dual Dipole Structure for UWB. IET Microw. Antennas Propag..

[11] Sor J., Deal W.R., Qian Y., Itoh T. A Broadband Quasi-Yagi Antenna Array. Proceedings of the 1999 29th European Microwave Conference.

[12] Qian Y., Deal W.R., Kaneda N., Itoh T. A uniplanar quasi-Yagi antenna with wide bandwidth and low mutual coupling characteristics. Proceedings of the IEEE Antennas and Propagation Society International Symposium.

[13] Kaneda N., Deal W.R., Qian Y., Waterhouse R., Itoh T. (2002). A Broad-Band Planar Quasi-Yagi Antenna. IEEE Trans. Antennas Propag..

[14] Sun M., Zhang Y.P. (2007). 100-GHz quasi-Yagi antenna in silicon technology. IEEE Electron Device Lett..

[15] Shao J., Fang G., Ji Y., Tan K., Yin H. (2013). A Novel Compact Tapered-Slot Antenna for GPR Applications. IEEE Antennas Wirel. Propag. Lett..

[16] Paulino H., Goes J., Garcao A.S. (2008). Low Power UWB CMOS Radar Sensors.

[17] (2015). X2 Impulse Radar Transceiver, XETHRU by NOVELDA.

[18] Baek I.S., Jung M.K., Cho S.H. (2013). Improvement of Computational Speed for the SVD Background Clutter Signal Subtraction Algorithm in IR-UWB Radar Systems. J. KICS.

[19] Kim B.H., Han S.J., Kwon G.R., Pyun J.Y. (2015). Signal Processing for Tracking of Moving Object in Multi-Impulse Radar Network System. Int. J. Distrib. Sens. Netw..

[20] Verma P.K., Gaikwad A.N., Singh D., Nigam M.J. (2009). Analysis of Clutter Reduction Techniques for Through Wall Imaging in UWB Range. Prog. Electromagn. Res..

[21] Singh S., Liang Q., Chen D., Sheng L. (2011). Sense through wall human detection using UWB radar. EURASIP J. Wirel. Commun. Netw..

[22] Hirata S., Kurosawa M.K., Katagiri T. (2008). Cross-Correlation by Single-bit Signal Processing for Ultrasonic Distance Measurement. IEICE Trans. Fundam. Electron. Commun. Comput. Sci..

[23] (2016). Vivaldi-LA Antenna Simula’on/Measrement Report.

[24] Wu J., Zhao Z., Nie Z., Liu Q.H. (2014). Design of a Wideband Planar Printed Quasi-Yagi Antenna Using Stepped Connection Structure. IEEE Trans. Antennas Propag..

[25] Yeo J., Lee J.I. (2016). Bandwidth Enhancement of Double-Dipole Quasi-Yagi Antenna Using Stepped Slotline Structure. IEEE Antennas Wirel. Propag. Lett..

